# Incidence of malignancy in adult patients with rheumatoid arthritis: a meta-analysis

**DOI:** 10.1186/s13075-015-0728-9

**Published:** 2015-08-15

**Authors:** Teresa A. Simon, Adam Thompson, Kunal K. Gandhi, Marc C. Hochberg, Samy Suissa

**Affiliations:** Bristol-Myers Squibb, Princeton, NJ USA; Departments of Medicine and Epidemiology and Public Health, University of Maryland School of Medicine, Baltimore, MD USA; Division of Clinical Epidemiology, McGill University, Montreal, QC Canada

## Abstract

**Introduction:**

Patients with rheumatoid arthritis (RA) are at an increased risk of malignancies compared with the general population. This has raised concerns regarding these patients, particularly with the widespread use of immunomodulating therapies, including biologic disease-modifying antirheumatic drugs (DMARDs). We performed a systematic literature review and analysis to quantify the incidence of malignancies in patients with RA and the general population to update previously published data.

**Methods:**

A literature search was conducted that was consistent with and similar to that in a meta-analysis published in 2008. MEDLINE, BIOSIS Previews, Embase, Derwent Drug File and SciSearch databases were searched using specified search terms. Predefined inclusion criteria identified the relevant observational studies published between 2008 and 2014 that provided estimates of relative risk of malignancy in patients with RA compared with the general population. Risk data on overall malignancy and site-specific malignancies (lymphoma, melanoma and lung, colorectal, breast, cervical and prostate cancer) were extracted. The standardized incidence ratios (SIRs; a measure of risk) relative to the general population were evaluated and compared with published rates.

**Results:**

A total of nine publications met the inclusion criteria. Seven of these reported SIRs for overall malignancy; eight for lymphoma, melanoma, and lung, colorectal and breast cancer; seven for prostate cancer; and four for cervical cancer. Compared with those in the general population, the SIR estimates for patients with RA suggest a modest increased risk in overall malignancy, as previously observed. Patients with RA continued to show an increased risk of lymphoma and lung cancer compared with the general population. Overall, SIR estimates for colorectal and breast cancers continued to show a decrease in risk, whereas cervical cancer, prostate cancer and melanoma appeared to show no consistent trend in risk among patients with RA compared with the general population.

**Conclusions:**

The additional data evaluated here are consistent with previously reported data. Patients with RA are at an increased risk of lung and lymphoma malignancies compared with the general population. Quantifying differences in malignancy rates between non-biologic and biologic DMARD-treated patients with RA may further highlight which malignancies may be related to treatment rather than to the underlying disease.

## Introduction

Rheumatoid arthritis (RA) is a polygenic, multifactorial and chronic immune-mediated disease characterized by chronic joint inflammation, a predilection for development of joint damage and deformity, and extraarticular involvement [1]. The management of RA includes the use of biologic and non-biologic disease-modifying antirheumatic drugs (DMARDs) [2, 3], which act by directly modifying immunologic pathways involved in the pathogenesis of RA. The risk of malignancy among patients with RA has been of ongoing interest and research because of the autoimmune pathogenesis that underlies RA, the common etiology between rheumatic disease and malignancy, and the use of immunomodulatory therapy, such as DMARDs, that may alter normal immunosurveillance and elevate the risk of malignancy [4, 5]. Understanding this potential therapeutic risk has become more relevant with the increasing use of biologic DMARDs as a routine therapeutic approach to RA management [3]. With more biologic treatment options available and initiation of biologic treatments occurring earlier, it is important to understand the baseline risk of malignancies in patients with RA. Furthermore, continuous updates on the incidence of malignancies published in the literature are crucial to allow better understanding of the background rates for malignancy in clinical trials and observational research evaluating real-world practice.

Smitten et al. [6] performed a meta-analysis on the risk of overall malignancy and several site-specific cancers, including overall lymphoma, Hodgkin disease, non-Hodgkin lymphoma, lung cancer, colorectal cancer and breast cancer, using data published between 1990 and 2007. Their meta-analysis suggested a small overall increase in risk of malignancy, which was elevated for lymphoma, Hodgkin disease, non-Hodgkin lymphoma and lung cancer, but they found a decreased risk of colorectal and breast cancer in patients with RA compared with the general population [6]. The malignancies included were prespecified and based on the most frequently reported malignancies in an RA population.

In this article, we review the data on the incidence of malignancy reported since the Smitten et al. [6] meta-analysis and additionally evaluate the risk of other important site-specific malignancies, namely, melanoma, cervical cancer and prostate cancer, which have been a topic of discussion in recent publications [7–9].

## Methods

### Literature search

We conducted a search of the MEDLINE, BIOSIS Previews, Embase, Derwent Drug File and SciSearch databases for literature published between 1 January 2008 and 30 November 2014, using the following specified search terms: *cancer* or *tumor*, *tumour*, malign* and *rheumatoid arthritis* or *RA*, and epidemiolog* or inciden*, *population*, *observation*, *retrospective* or occurren*.

### Exclusion algorithm

Predefined inclusion criteria were used to identify relevant observational studies that provided estimates of relative risk of malignancy in patients with RA compared with the general population. The same criteria as detailed by Smitten et al. [6] were applied. Studies were eligible for inclusion if they fulfilled the following criteria: (1) observational study design (including prospective, retrospective, epidemiologic, database, survey, registry, cohort and case–control), (2) reported malignancy outcomes in patients with RA and a general population, (3) enrolled more than 100 patients, (4) included only patients older than 18 years of age, (5) covered any geographic region and (6) were reported in English as a full-length publication. Citations meeting the inclusion criteria were obtained and screened for the outcomes of interest, which included the observed incidence rates of total malignancy and lymphoma, lung, colorectal, breast, melanoma, prostate and cervical cancers in patients with RA compared with the expected incidence rates in the general population. Lymphoma was reported as Hodgkin or non-Hodgkin where available. The selection of studies for inclusion was made without regard to evaluation of specific RA management strategies. We attempted to avoid overlap by excluding studies for which updated publications were available. We also excluded studies that compared specific RA biologic treatments with other RA treatments, unless overall RA and general population rates were reported.

### Data presentation

Citations meeting the inclusion criteria were screened for outcomes of interest. Risk data on overall malignancy and site-specific malignancies (overall lymphoma, Hodgkin disease, non-Hodgkin lymphoma, melanoma, and lung, colorectal, breast, cervical and prostate cancers) were extracted independently by two people and compared for accuracy. Study-specific estimates of relative risk were measured mostly by overall and age- and sex-adjusted standardized incidence ratios (SIRs) relative to non-RA patients or the general population. In one study using a case–control approach, researchers reported an odds ratio, which is an accurate estimator of the relative risk [10]. Thus, all measures of relative risk and their respective 95 % confidence intervals (CIs) were then compared with previously published rates. Pooled relative risks and 95 % CIs were then computed using the DerSimonian and Laird technique [11], in which a random-effects model takes into consideration both within-study and between-studies variation by incorporating the heterogeneity of the effects in the overall analysis. Some studies in which authors reported data separately for colon and rectal cancer, or for men and women, were considered separate studies when we pooled relative risks.

## Results

**Fig. 1:**
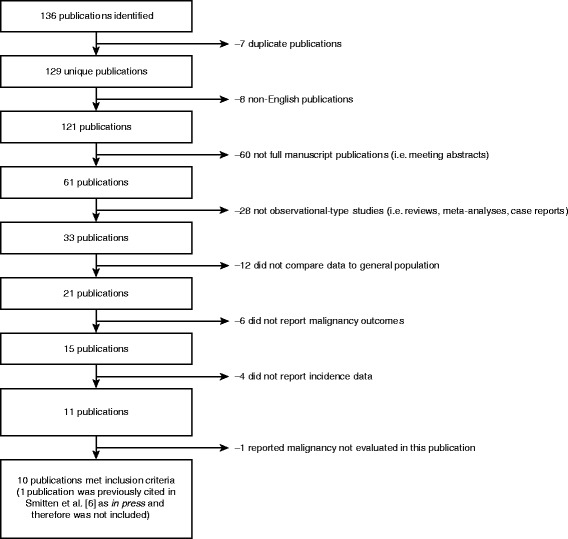
Literature search data

A total of 136 articles were identified using the defined search criteria (Fig. 1). Following identification, a total of 33 potential publications were further analyzed; of these, 9 studies met all the inclusion criteria (Fig. 1) [10, 12–19]. These studies included population- and community-based RA cohorts, examined data from 458 to 84,475 patients and had mean follow-up periods ranging from 4 to 25 years. The relative risk of overall malignancy was reported in seven studies [12–15, 17–19]; lymphoma (overall lymphoma, Hodgkin disease or non-Hodgkin lymphoma), lung, colorectal and breast cancers and melanoma in eight [10, 12–17, 19]; prostate cancer in seven [10, 12–17]; and cervical cancer in four [12, 14, 16, 17]. Overall risk by malignancy type was reported in eight studies [10, 12–17, 19], both overall risk and risk by sex were reported in three [15, 17, 18] and risk stratified by sex only was reported in one [16].

### Overall malignancies

**Fig. 2:**
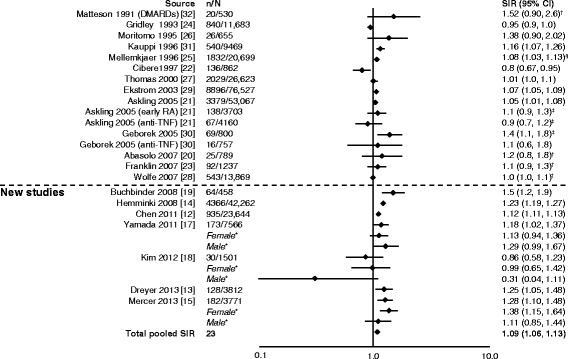
Relative risk of overall malignancy in patients with rheumatoid arthritis (RA) compared with the general population. CI, confidence interval; DMARD, disease-modifying antirheumatic drug; n, number of malignancies; N, population size; RR, relative risk; SIR, standardized incidence ratio; TNF, tumor necrosis factor. *SIRs by sex are not included in the total pooled SIR. ^†^Excluding non-melanoma skin cancer. ^‡^All solid tumors. ^§^Excluding lymphatic and hematopoietic

In general, SIRs for overall malignancy across the studies were similar. Six of seven studies reported a significant increase in overall risk of malignancy (Fig. 2) [12–15, 17–32]. The total pooled SIR (95 % CI) for studies analyzed by Smitten et al. (1.05 [1.01–1.09]) [6] and those included here was significant: 1.09 (1.06–1.13). With the addition of the newer studies, there was no change in the overall summary statistic showing that patients with RA have a 10 % increase in overall malignancy risk compared with the general population.

### Lymphoma

**Fig. 3:**
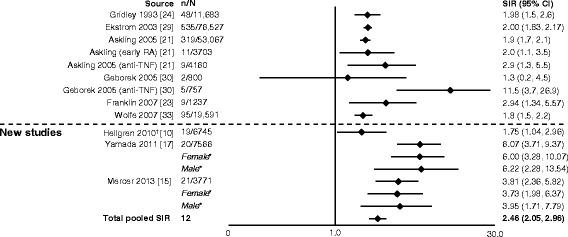
Relative risk of malignant lymphoma in patients with rheumatoid arthritis (RA) compared with the general population. CI, confidence interval; n, number of malignancies; N, population size; OR, odds ratio; SIR, standardized incidence ratio; TNF, tumor necrosis factor. *SIRs by sex are not included in the total pooled SIR. ^†^Reported as odds ratio

**Fig. 4:**
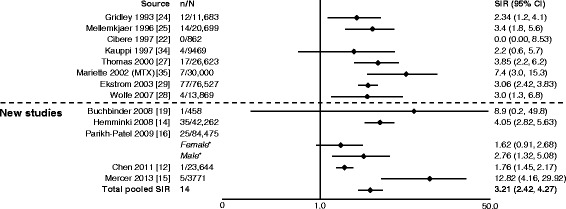
Relative risk of Hodgkin disease in patients with rheumatoid arthritis (RA) compared with the general population. CI, confidence interval; n, number of malignancies; N, population size; MTX, methotrexate; SIR, standardized incidence ratio. *SIRs by sex for Parikh-Patel et al. [16] are included in the total pooled SIR, as overall SIR was not available in their study

**Fig. 5:**
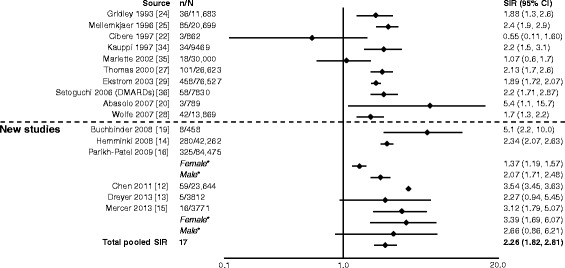
Relative risk of non-Hodgkin lymphoma in patients with rheumatoid arthritis (RA) compared with the general population. CI, confidence interval; DMARD, disease-modifying antirheumatic drug; n, number of malignancies; N, population size; SIR, standardized incidence ratio. *SIRs by sex included in total pooled SIR only if overall SIR was not available

In eight studies, authors reported outcomes as either overall lymphoma, Hodgkin disease or non-Hodgkin lymphoma [10, 12–17, 19]. Two studies reported only lymphoma, whereas the remaining six studies reported overall lymphoma, Hodgkin disease and non-Hodgkin lymphoma rates, with all of the new studies reporting a significant increase in risk of lymphoma in patients with RA compared with the general population (Figs. 3, 4 and 5). This ranged from almost a doubling (SIR 1.75) (Fig. 3) [10, 15, 17, 21, 23, 24, 29, 30, 33] to a 12-fold increase (SIR 12.82) (Fig. 4) [12, 14–16, 19, 22, 24, 25, 27–29, 34, 35] in the risk of developing lymphoma. The pooled SIR for Hodgkin disease (Fig. 4) was higher than for non-Hodgkin lymphoma (Fig. 5) [12–16, 19, 20, 22, 24, 25, 27–29, 34–36]. The total pooled SIR (95 % CI) for the studies reviewed here and by Smitten et al. [6] was 2.46 (2.05–2.96) for malignant lymphoma, 3.21 (2.42–4.27) for Hodgkin disease and 2.26 (1.82–2.81) for non-Hodgkin lymphoma. The overall risk with the addition of the new studies is consistent with previously reported SIRs (2.08 [1.80–2.39], 3.29 [2.56–4.22] and 1.95 [1.70–2.24], respectively) [6]; however, a slight increase in risk for non-Hodgkin disease was noted.

### Lung cancer

**Fig. 6:**
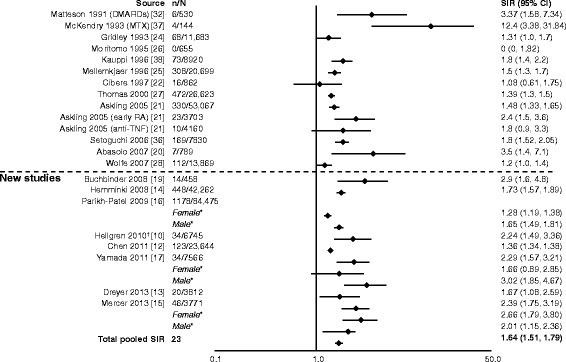
Relative risk of lung cancer in patients with rheumatoid arthritis (RA) compared with the general population. CI, confidence interval; DMARD, disease-modifying antirheumatic drug; MTX, methotrexate; n, number of malignancies; N, population size; SIR, standardized incidence ratio; TNF, tumor necrosis factor. *SIRs by sex are included in total pooled SIR only if overall SIR was not available. ^†^Reported as odds ratio

In eight studies, authors reported that patients with RA had an increased risk of lung cancer compared with the general population [10, 12–17, 19]. The SIRs (95 % CI) for lung cancer ranged from 1.36 (1.34–1.38) to 2.9 (1.6–4.8) (Fig. 6) [10, 12–17, 19–22, 24–28, 32, 36–38]. The total pooled SIR (95 % CI) for the studies reviewed here and by Smitten et al. [6] was 1.64 (1.51–1.79) for lung cancer. The risk of lung cancer in patients with RA compared with the general population in the contemporary study is consistent with what was previously observed (1.63 [1.43–1.87]) [6].

### Colorectal cancer

**Fig. 7:**
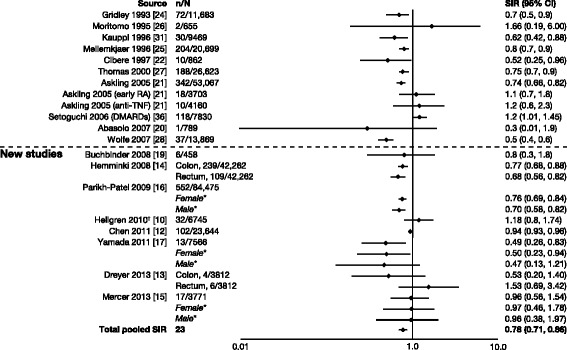
Relative risk of colorectal cancer in patients with rheumatoid arthritis (RA) compared with the general population. CI, confidence interval; DMARD, disease-modifying antirheumatic drug; n, number of malignancies; N, population size; SIR, standardized incidence ratio; TNF, tumor necrosis factor. *SIRs by sex are included in total pooled SIR only if overall SIR was not available. ^†^Reported as odds ratio

In seven studies [12, 14–17, 19], authors reported that patients with RA had a decreased risk of colon or colorectal cancer, with SIRs (95 % CI) ranging from 0.49 (0.26–0.83) to 0.96 (0.56–1.54) (Fig. 7) [10, 12–17, 19–22, 24–28, 31, 36]. However, in some studies, authors reported SIRs >1, with the highest being 1.53 (0.69–3.42) for rectal cancer [10, 13]. For colorectal cancer, the total pooled SIR (95 % CI) for the studies reviewed by Smitten et al. (0.77 [0.65–0.90]) [6] plus those included here was 0.78 (0.71–0.86).

### Breast cancer

**Fig. 8:**
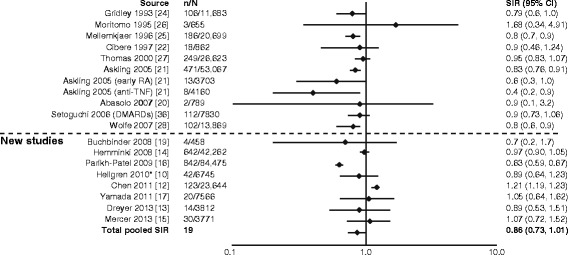
Relative risk of breast cancer in patients with rheumatoid arthritis (RA) compared with the general population. CI, confidence interval; DMARD, disease-modifying antirheumatic drug; n, number of malignancies; N, population size; SIR, standardized incidence ratio; TNF, tumor necrosis factor. *Reported as odds ratio

For breast cancer, there was generally no increase in risk; estimated risk varied from 0.63 to 1.21 in the new studies [10, 12–17, 19] (Fig. 8) [10, 12–17, 19–22, 24–28, 36]. The total pooled SIR (95 % CI) for the studies reviewed by Smitten et al. (0.84 [0.79–0.90]) [6] plus those included here was found to be 0.86 (0.73–1.01) for breast cancer.

### Melanoma and cervical and prostate cancers

**Fig. 9:**
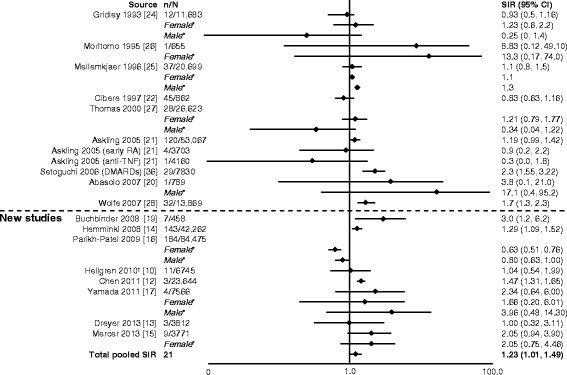
Relative risk of melanoma in patients with rheumatoid arthritis (RA) compared with the general population. CI, confidence interval; DMARD, disease-modifying antirheumatic drug; n, number of malignancies; N, population size; SIR, standardized incidence ratio; TNF, tumor necrosis factor. *SIRs by sex are included in total pooled SIR only if overall SIR was not available. ^†^Reported as odds ratio

**Fig. 10:**
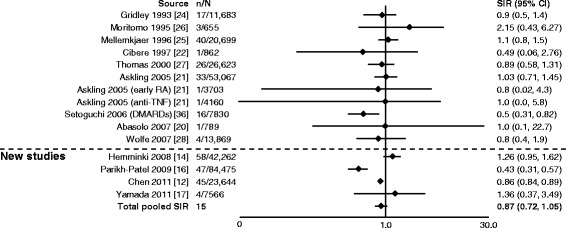
Relative risk of cervical cancer in patients with rheumatoid arthritis (RA) compared with the general population. CI, confidence interval; DMARD, disease-modifying antirheumatic drug; n, number of malignancies; N, population size; SIR, standardized incidence ratio; TNF, tumor necrosis factor

**Fig. 11:**
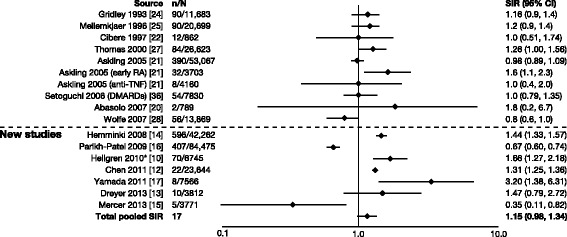
Relative risk of prostate cancer in patients with rheumatoid arthritis (RA) compared with the general population. CI, confidence interval; n, number of malignancies; N, population size; SIR, standardized incidence ratio. *Reported as odds ratio

As previously noted, we included three additional malignancies (melanoma and cervical and prostate cancer) in our assessment that were not included in the Smitten et al. meta-analysis [6]. Figs. 9, 10 and 11 highlight the articles analyzed by Smitten et al. [6] and the newer studies included in our assessment. For melanoma, we reviewed 17 studies (9 from Smitten et al. [20–22, 24–28, 36] and 8 new [10, 12–17, 19]). Of these, the overall range of SIRs was 0.3–8.83, and five studies [12, 14, 19, 28, 36] showed a significant increase in the risk of melanoma in the RA population, ranging from 1.29 to 3.0 (Fig. 9) [10, 12–17, 19–22, 24–28, 36]. The total pooled SIR was 1.23 (1.01–1.49).

For cervical cancer, SIRs were reported in a total of 13 studies (nine from Smitten et al. [20–22, 24–28, 36] and four new [12, 14, 16, 17]). Overall, the range of SIRs was 0.43–2.15, and all confidence intervals were overlapping (Fig. 10) [12, 14, 16, 17, 20–22, 24–28, 36]. In three studies, authors reported a significant decrease in risk compared with the RA population [12, 16, 36]. The total pooled SIR for cervical cancer was 0.87 (0.72–1.05). For prostate cancer, SIRs were reported in a total of 15 studies (8 from Smitten et al. [20–22, 24, 25, 27, 28, 36] and 7 new [10, 12–17]). Overall, the range of SIRs was 0.35–3.20, and the total pooled SIR was 1.15 (0.98–1.34) (Fig. 11) [10, 12–17, 20–22, 24, 25, 27, 28, 36].

## Discussion

Although individual studies can show considerable variation, the results of this updated meta-analysis of the incidence of malignancy in adult patients with RA are generally consistent with previously reported data for all specific malignancies [6]. The updated data reported here show that patients with RA have a modest increased risk of overall malignancy as well as an increased risk of lung cancer and lymphoma when compared with the general population. Lymphoma was associated with the greatest relative risk, especially Hodgkin disease, with a 12-fold increase reported in one study [15] and an increase in women versus men in another [16]. Lung cancer generally showed a twofold increase in risk [10, 12–17, 19]. As previously noted, a decreased risk overall of colorectal cancer in patients with RA compared with the general population was found. For melanoma, the pooled SIR was significantly greater than unity, although few of the individual point estimates were statistically significant. Breast and cervical cancers in general showed a decrease in risk overall that failed to reach statistical significance.

Various explanations for the differences observed in the risk of certain malignancies in patients with RA have been proposed. It is thought that ongoing immunologic stimulation over time may increase the risk of malignant transformation of immune system cells and decrease the number of T-suppressor lymphocytes, thus increasing rates of lymphoma malignancy in patients with RA [4]. Chronic lung inflammation due to the disease in patients with RA may explain the increased risk of lung cancer. Smoking increases the risk of both RA and lung cancer; however, the SIRs reported here were not adjusted for smoking, so it is not possible to rule out the increase in SIR being due to an indirect association. Thus, the risk of both lymphoma and lung cancer has been hypothesized to be dependent on the level of disease activity experienced by the patient [4, 6].

Patients with RA continued to show no increase in overall risk of colorectal and breast cancers, in contrast to their increased risk for lung cancer and lymphoma. In patients with RA, any observed decreased risk in colorectal malignancy may be due to the increased use of non-steroidal anti-inflammatory drugs, which are known to decrease this risk [4, 6]. Overall, the risk of malignancy among patients with RA may be due in part to the autoimmune pathogenesis of RA and common etiology between RA and malignancy, including genetic factors, smoking-related tissue necrosis and viral infection [9]. The three other site-specific malignancies examined here but not studied previously by Smitten et al. [6]—prostate cancer, cervical cancer and melanoma—are all common cancers, with cervical cancer being attributable to viral infection. In the present analysis, there did not appear to be a consistent trend of increased or decreased risk of prostate cancer, cervical cancer or melanoma among patients with RA compared with the general population. One review published in 2008 that did not meet our criteria showed melanoma results that were consistent with our present assessment. Some populations showed an increase in risk, whereas others did not [8].

This review, like that of Smitten et al. [6], is based on observational, real-world clinical data; however, these individual studies may have potential limitations. Although most of the individual estimates were derived from similar database studies, their study designs (including populations, data sources, follow-up times, data analysis and procedures of reporting, particularly when linked to a cancer registry) were a source of heterogeneity among the studies and may be associated with bias of varying magnitude. In addition, other potential sources of heterogeneity included geography and/or region, stage and severity of RA, and treatment. Such heterogeneity should be considered in any meta-analysis; the DerSimonian and Laird technique used in this study [11] assumes heterogeneity across studies and incorporates it into the estimation of the pooled SIRs and their confidence limits. Therefore, although the variation observed among the individual SIRs reported should be treated with caution when interpreting these data, it should be recognized that this variation was accounted for in the analyses resulting in the pooled SIRs.

For example, with regard to treatment, Dreyer et al. [13] evaluated the effect of tumor necrosis factor (TNF) inhibitor treatment on risk rate by examining the treated and non-treated populations separately (only non-treated data included in this analysis), whereas Mercer et al. [15] evaluated biologic-naive patients. Buchbinder et al. [19] examined malignancy risk only in patients with RA receiving methotrexate. However, the rest of the patients in these studies are likely to have received treatments for RA, therefore making it difficult to differentiate the effects of treatment and disease. Nonetheless, to further understand which malignancies may be related to treatment versus the underlying disease, it is important, where possible, to quantify differences in malignancy rates between patients with RA treated with non-biologic DMARDs and those treated with biologic DMARDs. Whether biologic agents, predominantly TNF inhibitors, increase the risk of malignancy has been debated [9]. Data have shown that use of TNF inhibitors was not associated with a major further increase in risk in the already elevated lymphoma occurrence in patients with RA [39–44]. However, they do appear to increase the risk of skin cancer, including melanoma [39–43]. Furthermore, the data presented by Simon et al. suggest that the observed incidence of total malignancy and those for breast, colorectal and lung cancers and lymphoma in patients treated with abatacept was largely consistent with that expected of patients with RA treated with non-biologic DMARDs [45].

## Conclusions

The additional data presented here are consistent with previously reported data [6] for all specified malignancies. For overall malignancy, the new studies analyzed here show a similar increase in risk compared with that previously reported. In addition, patients with RA continue to be at an increased risk of lung cancer and lymphoma when compared with the general population. Further studies examining specific aspects such as treatments, smoking or other lifestyle factors are needed to investigate the underlying mechanisms for the increased or decreased risk of specific cancers observed in patients with RA compared with the general population. Also, it is important to quantify any differences in malignancy risks between patients with RA who are treated with non-biologic DMARDs and those treated with biologic DMARDs, as understanding which malignancies may be related to the treatment as opposed to being related to underlying disease will allow patients to be advised and monitored accordingly.

## Abbreviations

CI: confidence interval
DMARD: disease-modifying anti-rheumatic drug
MTX: methotrexate
NR: not reported
OR: odds ratio
RA: rheumatoid arthritis
RR: relative risk
SIR: standardized incidence ratio
TNF: tumor necrosis factor

## References

[1] Aletaha D, Neogi T, Silman AJ, Funovits J, Felson DT, Bingham CO (2010). 2010 rheumatoid arthritis classification criteria: an American College of Rheumatology/European League Against Rheumatism collaborative initiative. Arthritis Rheum.

[2] Smolen JS, Landewé R, Breedveld FC, Dougados M, Emery P, Gaujoux-Viala C (2010). EULAR recommendations for the management of rheumatoid arthritis with synthetic and biological disease-modifying antirheumatic drugs. Ann Rheum Dis.

[3] Singh JA, Furst DE, Bharat A, Curtis JR, Kavanaugh AF, Kremer JM (2012). 2012 update of the 2008 American College of Rheumatology recommendations for the use of disease-modifying antirheumatic drugs and biologic agents in the treatment of rheumatoid arthritis. Arthritis Care Res (Hoboken).

[4] Love T, Solomon DH (2008). The relationship between cancer and rheumatoid arthritis: still a large research agenda. Arthritis Res Ther.

[5] Cush JJ, Dao KH (2012). Malignancy risks with biologic therapies. Rheum Dis Clin North Am.

[6] Smitten AL, Simon TA, Hochberg MC, Suissa S (2008). A meta-analysis of the incidence of malignancy in adult patients with rheumatoid arthritis. Arthritis Res Ther.

[7] Kim SC, Glynn RJ, Giovannucci E, Hernández-Díaz S, Liu J, Feldman S (2015). Risk of high-grade cervical dysplasia and cervical cancer in women with systemic inflammatory diseases: a population-based cohort study. Ann Rheum Dis.

[8] Chakravarty EF, Farmer ER (2008). Risk of skin cancer in the drug treatment of rheumatoid arthritis. Expert Opin Drug Saf.

[9] Szekanecz Z, Szekanecz É, Bakó G, Shoenfeld Y (2011). Malignancies in autoimmune rheumatic disease – a mini-review. Gerontology.

[10] Hellgren K, Smedby KE, Feltelius N, Baecklund E, Askling J (2010). Do rheumatoid arthritis and lymphoma share risk factors? A comparison of lymphoma and cancer risks before and after diagnosis of rheumatoid arthritis. Arthritis Rheum.

[11] DerSimonian R, Laird N (1986). Meta-analysis in clinical trials. Control Clin Trials.

[12] Chen YJ, Chang YT, Wang CB, Wu CY (2011). The risk of cancer in patients with rheumatoid arthritis: a nationwide cohort study in Taiwan. Arthritis Rheum.

[13] Dreyer L, Mellemkjær L, Andersen AR, Bennett P, Poulsen UE, Juulsgaard Elligsen T (2013). Incidences of overall and site specific cancers in TNFα inhibitor treated patients with rheumatoid arthritis and other arthritides – a follow-up study from the DANBIO Registry. Ann Rheum Dis.

[14] Hemminki K, Li X, Sundquist K, Sundquist J (2008). Cancer risk in hospitalized rheumatoid arthritis patients. Rheumatology.

[15] Mercer L, Davies R, Galloway JB, Low A, Lunt M, Dixon WG (2013). Risk of cancer in patients receiving non-biologic disease-modifying therapy for rheumatoid arthritis compared with the UK general population. Rheumatology (Oxford).

[16] Parikh-Patel A, White RH, Allen M, Cress R (2009). Risk of cancer among rheumatoid arthritis patients in California. Cancer Causes Control.

[17] Yamada T, Nakajima A, Inoue E, Tanaka E, Taniguchi A, Momohara S (2011). Incidence of malignancy in Japanese patients with rheumatoid arthritis. Rheumatol Int.

[18] Kim YJ, Shim JS, Choi CB, Bae SC (2012). Mortality and incidence of malignancy in Korean patients with rheumatoid arthritis. J Rheumatol.

[19] Buchbinder R, Barber M, Heuzenroeder L, Wluka AE, Giles G, Hall S (2008). Incidence of melanoma and other malignancies among rheumatoid arthritis patients treated with methotrexate. Arthritis Rheum.

[20] Abásolo L, Júdez E, Descalzo MÁ, González-Álvaro I, Jover JA, Carmona L, EMECAR Study Group (2008). Cancer in rheumatoid arthritis: occurrence, mortality and associated factors in a South European population. Semin Arthritis Rheum.

[21] Askling J, Fored CM, Brandt L, Baecklund E, Bertilsson L, Feltelius N (2005). Risks of solid cancers in patients with rheumatoid arthritis and after treatment with tumour necrosis factor antagonists. Ann Rheum Dis.

[22] Cibere J, Sibley J, Haga M (1997). Rheumatoid arthritis and the risk of malignancy. Arthritis Rheum.

[23] Franklin J, Lunt M, Bunn D, Symmons D, Silman A (2007). Influence of inflammatory polyarthritis on cancer incidence and survival: results from a community-based prospective study. Arthritis Rheum.

[24] Gridley G, McLaughlin JK, Ekbom A, Klareskog L, Adami HO, Hacker DG (1993). Incidence of cancer among patients with rheumatoid arthritis. J Natl Cancer Inst.

[25] Mellemkjaer L, Linet MS, Gridley G, Frisch M, Møller H, Olsen JH (1996). Rheumatoid arthritis and cancer risk. Eur J Cancer.

[26] Moritomo H, Ueda T, Hiyama T, Hosono N, Mori S, Komatsubara Y (1995). The risk of cancer in rheumatoid patients in Japan. Scand J Rheumatol.

[27] Thomas E, Brewster DH, Black RJ, Macfarlane GJ (2000). Risk of malignancy among patients with rheumatic conditions. Int J Cancer.

[28] Wolfe F, Michaud K (2007). Biologic treatment of rheumatoid arthritis and the risk of malignancy: analyses from a large US observational study. Arthritis Rheum.

[29] Ekström K, Hjalgrim H, Brandt L, Baecklund E, Klareskog L, Ekbom A (2003). Risk of malignant lymphomas in patients with rheumatoid arthritis and in their first-degree relatives. Arthritis Rheum.

[30] Geborek P, Bladström A, Turesson C, Gulfe A, Petersson IF, Saxne T (2005). Tumour necrosis factor blockers do not increase overall tumour risk in patients with rheumatoid arthritis, but may be associated with an increased risk of lymphomas. Ann Rheum Dis.

[31] Kauppi M, Pukkala E, Isomaki H (1996). Low incidence of colorectal cancer in patients with rheumatoid arthritis. Clin Exp Rheumatol.

[32] Matteson EL, Hickey AR, Maguire L, Tilson HH, Urowitz MB (1991). Occurrence of neoplasia in patients with rheumatoid arthritis enrolled in a DMARD Registry. Rheumatoid Arthritis Azathioprine Registry Steering Committee. J Rheumatol.

[33] Wolfe F, Michaud K (2007). The effect of methotrexate and anti-tumor necrosis factor therapy on the risk of lymphoma in rheumatoid arthritis in 19,562 patients during 89,710 person-years of observation. Arthritis Rheum.

[34] Kauppi M, Pukkala E, Isomäki H (1997). Elevated incidence of hematologic malignancies in patients with Sjögren’s syndrome compared with patients with rheumatoid arthritis (Finland). Cancer Causes Control.

[35] Mariette X, Cazals-Hatem D, Warszawki J, Liote F, Balandraud N, Sibilia J, the Club Rhumatismes et Inflammation investigators (2002). Lymphomas in rheumatoid arthritis patients treated with methotrexate: a 3-year prospective study in France. Blood.

[36] Setoguchi S, Solomon DH, Weinblatt ME, Katz JN, Avorn J, Glynn RJ (2006). Tumor necrosis factor α antagonist use and cancer in patients with rheumatoid arthritis. Arthritis Rheum.

[37] McKendry RJ, Dale P (1993). Adverse effects of low dose methotrexate therapy in rheumatoid arthritis. J Rheumatol.

[38] Kauppi M, Pukkala E, Isomäki H (1996). Excess risk of lung cancer in patients with rheumatoid arthritis. J Rheumatol.

[39] Mariette X, Matucci-Cerinic M, Pavelka K, Taylor P, van Vollenhoven R, Heatley R (2011). Malignancies associated with tumour necrosis factor inhibitors in registries and prospective observational studies: a systematic review and meta-analysis. Ann Rheum Dis.

[40] Park HJ, Ranganathan P (2012). TNF-α antagonism and cancer risk in rheumatoid arthritis: is continued vigilance warranted?. Discov Med.

[41] Le Blay P, Mouterde G, Barnetche T, Morel J, Combe B (2012). Risk of malignancy including non-melanoma skin cancers with anti-tumor necrosis factor therapy in patients with rheumatoid arthritis: a meta-analysis of registries and systematic review. Clin Exp Rheumatol.

[42] Askling J, Fahrbach K, Nordstrom B, Ross S, Schmid CH, Symmons D (2011). Cancer risk with tumor necrosis factor α (TNF) inhibitors: meta-analysis of randomized controlled trials of adalimumab, etanercept, and infliximab using patient level data. Pharmacoepidemiol Drug Saf.

[43] Damjanov N, Nurmohamed MT, Szekanecz Z (2014). Biologics, cardiovascular effects and cancer. BMC Med.

[44] Askling J, Baecklund E, Granath F, Geborek P, Fored M, Backlin C (2009). Anti-tumour necrosis factor therapy in rheumatoid arthritis and risk of malignant lymphomas: relative risks and time trends in the Swedish Biologics Register. Ann Rheum Dis.

[45] Simon TA, Smitten AL, Franklin J, Askling J, Lacaille D, Wolfe F (2009). Malignancies in the rheumatoid arthritis abatacept clinical development programme: an epidemiological assessment. Ann Rheum Dis.

