# Erratum to: Incidence of malignancy in adult patients with rheumatoid arthritis: a meta-analysis

**DOI:** 10.1186/s13075-016-0990-5

**Published:** 2016-05-04

**Authors:** Teresa A. Simon, Adam Thompson, Kunal K. Gandhi, Marc C. Hochberg, Samy Suissa

**Affiliations:** Bristol-Myers Squibb, Princeton, NJ USA; Departments of Medicine and Epidemiology and Public Health, University of Maryland School of Medicine, Baltimore, MD USA; Division of Clinical Epidemiology, McGill University, Montreal, QC Canada

It has come to our attention that, in our recently published article on the incidence of malignancy in adult patients with rheumatoid arthritis [1], the risk of melanoma requires additional clarification. Indeed, the meta-analysis of the risk of melanoma among patients with rheumatoid arthritis compared with the general population showed a standardized incidence rate ratio of 1.23 (95 % confidence interval 1.01, 1.49). This finding was erroneously included in the list of malignancies for which there was no consistent trend in risk among patients with rheumatoid arthritis versus the general population. Instead, with our current, updated meta-analysis it should be considered that there is now a suggestion of a possible increased risk for melanoma. With respect to the Discussion section of our article, we would like to note that the summary standardized incidence ratio shows a modest increased risk for melanoma.
